# Patterns, Prevalence, and Outcomes of Pediatric Trauma: A Cross-Sectional Study From South India

**DOI:** 10.7759/cureus.95732

**Published:** 2025-10-30

**Authors:** Harikrishnan Elangovan, Pranav Munnuswamy

**Affiliations:** 1 Pediatrics, Government Villupuram Medical College, Villupuram, IND

**Keywords:** burns, falls, injury outcomes, pediatric trauma, road traffic accidents, south india, trauma prevention

## Abstract

Background: Pediatric trauma remains a leading cause of morbidity and mortality worldwide, yet many regions in India still lack comprehensive data.

Objective: We evaluated the demographic and clinical characteristics, mechanisms, and outcomes of pediatric trauma in Villupuram, South India.

Methods: We conducted a six-month prospective study at a tertiary center in Villupuram, enrolling trauma patients under 18 years using purposive sampling. Exclusions included poisoning, bites, chronic conditions, psychiatric cases, and patients over 18. Data were collected via a structured proforma, with parental consent obtained. Patients were stratified into four age groups. Analysis was done using IBM SPSS Statistics for Windows, Version 29.0 (IBM Corp., Armonk, New York, United States), with chi-squared tests (p<0.05 significant).

Results: We included 438 pediatric trauma cases. Males accounted for 71% of cases, with a mean age of 11.3 years. Roads represented the most frequent site of injury (63%). Two-wheeler accidents (29.5%), burns (27.4%), and falls from height (20.1%) were the leading mechanisms of trauma. Abrasions (41.6%) and lacerations (30.1%) were the most common injuries, and the upper limb, head, and lower limb were the most frequently affected anatomical sites.

Conclusion: Pediatric trauma in South India is dominated by road traffic accidents, burns, and falls, with adolescent males disproportionately affected. These findings highlight the urgent need for targeted prevention, including road safety enforcement, burn prevention strategies, safer housing design, and strengthened trauma systems with long-term rehabilitation.

## Introduction

Trauma occurs when external mechanical forces injure the body. Motor vehicle accidents, falls, interpersonal violence, and burns commonly cause such injuries. These events may result in outcomes ranging from localized damage to widespread physiological disruption and psychological distress [1].

Pediatric trauma contributes substantially to global morbidity and mortality, claiming millions of lives and disability-adjusted life years (DALYs) each year. Southeast Asia bears a disproportionate share of this burden [2]. However, India still lacks a national pediatric trauma registry, and limited epidemiological data constrain the development of targeted prevention and management strategies [3].

In this study, we analyzed the demographic and clinical profiles of pediatric trauma cases in the Villupuram region. We identified the most common mechanisms of injury, assessed outcomes, and aimed to provide evidence for preventive interventions tailored to local needs.

## Materials and methods

We conducted a prospective descriptive study at Government Villupuram Medical College and Hospital in Villupuram, India, over a six-month period (January-July 2023). We included all pediatric patients under 18 years of age admitted with trauma. We excluded patients older than 18 years and cases involving poisoning, animal or insect bites, chronic medical conditions, or psychiatric disorders.

We used purposive sampling to enroll eligible participants, ensuring the comprehensive capture of trauma cases during the study period, given the unpredictable nature of emergency admissions. This approach was chosen to avoid the underrepresentation of high-acuity cases and to reflect real-world emergency department flow. Emergency room (ER) nurses trained in documentation and standardized trauma assessment protocols collected data using a structured proforma. Training sessions included mock documentation exercises, supervised pilot entries, and feedback from senior clinicians to ensure consistency and reduce inter-observer variability. Before full-scale data collection, we pilot-tested the proforma on 20 cases to ensure clarity and consistency. The tool captured demographic details, sex, place and mode of injury, anatomical site, symptomatology, radiological investigations, specialty consultations, therapeutic interventions, duration of hospital stay, and clinical outcomes.

Primary outcomes assessed included injury severity (simple vs. grievous), type of intervention (conservative vs. surgical), and discharge status (e.g., discharged, referred, discharged at request, or against medical advice). These were selected to reflect short-term clinical status and resource utilization. We excluded incomplete records from analysis and handled missing variables using pairwise deletion.

To improve diagnostic precision, we defined "type of injury" as the systemic trauma diagnosed clinically (e.g., orthopedic injury, burn, polytrauma) and "type of wound" as the external soft tissue manifestations observed on initial examination (e.g., abrasion, laceration, contusion). We recorded these categories separately to avoid overlap.

We stratified patients into four age groups: infants and toddlers (0-3 years), preschoolers (4-6 years), young school-aged children (7-9 years), and adolescents (10-18 years). We analyzed the data using IBM SPSS Statistics for Windows, Version 29.0 (IBM Corp., Armonk, New York, United States), and applied the chi-squared test to compare categorical variables across age groups (e.g., sex distribution, injury type). We considered a p-value of <0.05 statistically significant.

This study was approved by the Institutional Ethics Committee of Government Villupuram Medical College (approval number: GVMC/IEC/2023/09) on January 9, 2023.

## Results

**Figure 1:**
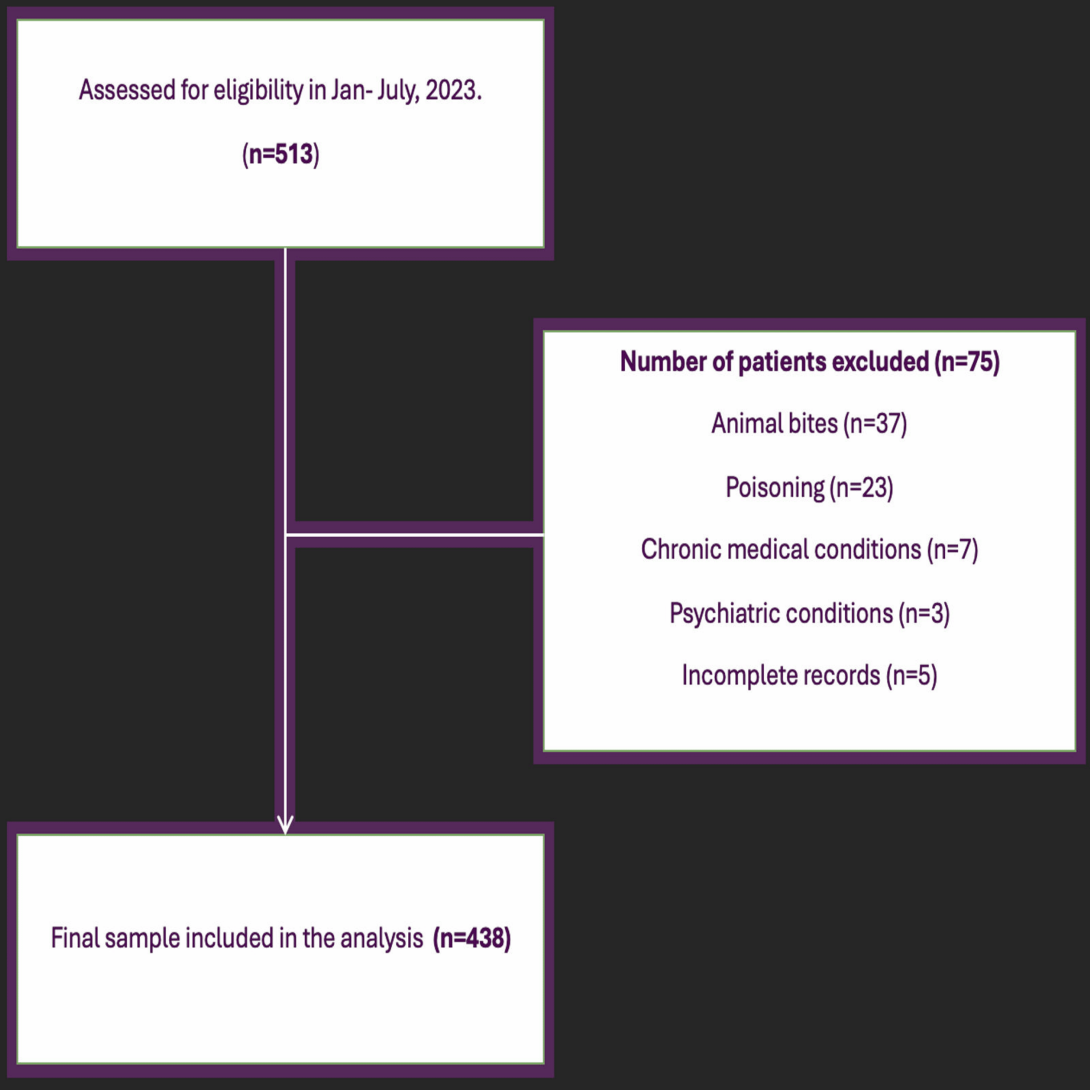
STROBE flowchart of patient selection Exclusion criteria applied as per study protocol. Incomplete records excluded from analysis. STROBE: Strengthening the Reporting of Observational Studies in Epidemiology

We identified 438 pediatric trauma cases that met the inclusion criteria during the six-month study period. Figure 1 presents the Strengthening the Reporting of Observational Studies in Epidemiology (STROBE)-compliant flowchart of patient selection, detailing exclusions and final sample size.

**Figure 2:**
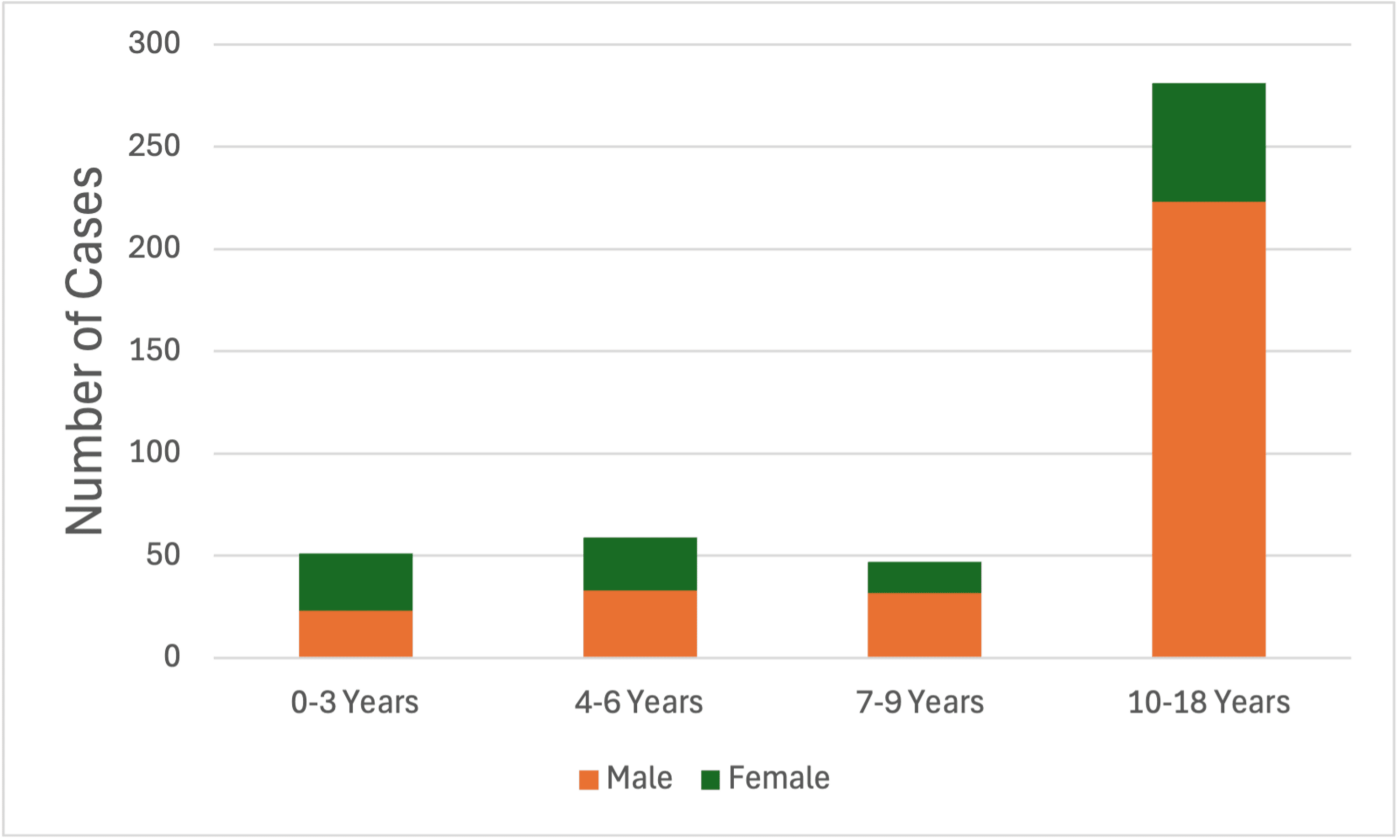
Distribution of sex across pediatric age groups Stacked bar chart depicting the number of male (orange) and female (green) pediatric trauma cases across four age categories: 0-3 years, 4-6 years, 7-9 years, and 10-18 years. Male cases consistently outnumber female cases in each age group, with the highest overall case volume observed in the 10-18-year category.

Of these, 311 (71%) were males and 127 (29%) were females, with a mean age of 11.36±0.26 years. We stratified patients into four age groups and observed a consistent male predominance across all categories, especially among adolescents. Table 1 presents the age-stratified demographic and clinical characteristics, while Figure 2 illustrates the distribution of sex across pediatric age groups.

**Table 1:** Age-stratified distribution of demographic, clinical, and injury characteristics in the study population (n=438) This table presents pediatric trauma data categorized by age groups: 0-3 years, 4-6 years, 7-9 years, and 10-18 years. Variables include sex distribution, injury severity ("hurt"), duration of hospital stay, discharge status, mode and site of injury, type of injury, and type of wound. Chi-squared (χ²) values and p-values are provided to assess statistical significance across age groups. *Injury type was recorded only for cases with definitive classification, excluding patients with ambiguous or undocumented injuries. **Multiple injury sites were documented per patient, resulting in a cumulative count exceeding the total number of individuals.

	Age group				Total (%)	χ^2^	P-value
	0-3 years	4-6 years	7-9 years	10-18 years			
Sex distribution							
Male	23	33	32	223	311 (71%)	32.8576	<0.001
Female	28	26	15	58	127 (29%)		
Total	51	59	47	281	438		
Hurt							
Simple	38	32	28	177	275 (62.79%)	5.0579	0.167
Grievous	13	27	19	104	163 (37.21%)		
Total	51	59	47	281	438		
Length of stay							
1 hour	15	18	11	76	120 (27.4%)	13.006625	0.601
6-12 hours	7	8	4	32	51 (11.64%)		
12-24 hours	18	11	14	91	134 (30.59%)		
1-3 days	6	13	12	40	71 (16.21%)		
3-5 days	3	3	4	24	34 (7.76%)		
5 days	2	6	2	18	28 (6.39%)		
Total	51	59	47	281	438		
Outcome							
Discharged	23	34	19	171	247 (56.39%)	14.2345	0.114
Discharged at request	24	19	19	83	145 (33.11%)		
Referral	3	4	7	23	37 (8.45%)		
Against medical advice	1	2	2	4	9 (2.05%)		
Total	51	59	47	281	438		
Mode of injury							
Fall	10	20	11	47	88 (20.09%)	40.096309	<0.001
Pedestrian	12	5	8	31	56 (12.79%)		
Two-wheeler	11	16	9	93	129 (29.45%)		
Four-wheeler	5	8	6	19	38 (8.68%)		
Burns	12	8	10	90	120 (27.4%)		
Sports	1	2	3	1	7 (1.6%)		
Total	51	59	47	281	438		
Place							
Home	21	22	10	65	118 (26.94%)	4.807226	0.85
Road	26	32	29	189	276 (63.01%)		
Farm	1	1	4	11	17 (3.88%)		
School/day care	2	2	3	11	18 (4.11%)		
Playground	1	2	1	5	9 (2.05%)		
Total	51	59	47	281	438		
Type of injury							
Orthopedic	6	12	16	60	94 (47.96%)	37.299492	0.04
Head	2	3	5	12	22 (11.22%)		
Chest	1	3	1	2	7 (3.57%)		
Abdomen	2	4	1	2	9 (4.59%)		
Burn	6	3	1	17	27 (13.78%)		
Polytrauma	2	3	3	5	13 (6.63%)		
Genital	1	1	2	3	7 (3.57%)		
Scald	3	5	1	1	10 (5.1%)		
Crush injury	1	1	2	3	7 (3.57%)		
Total	24	35	32	105	196*		
Site of injury							
Head	20	22	19	99	160 (21.86%)	37.482003	0.039
Face	21	27	15	81	144 (19.67%)		
Back	4	1	2	5	12 (1.64%)		
Chest	6	4	6	26	42 (5.74%)		
Upper limb	15	14	16	122	167 (22.81%)		
Abdomen	3	2	3	10	18 (2.46%)		
Pelvis	2	1	5	23	31 (4.23%)		
Lower limb	8	19	10	115	152 (20.77%)		
Polytrauma	1	1	1	3	6 (0.82%)		
Total	80	91	77	484	732**		
Type of wounds							
Abrasion	22	20	17	123	182 (41.55%)	9.626441	0.842
Laceration	10	17	16	89	132 (30.14%)		
Puncture wounds	1	1	1	2	5 (1.14%)		
Avulsion	2	1	1	7	11 (2.51%)		
Contusion	8	10	9	35	62 (14.16%)		
Swelling	2	6	7	31	46 (10.5%)		
Total	45	55	51	287	438		

**Figure 3:**
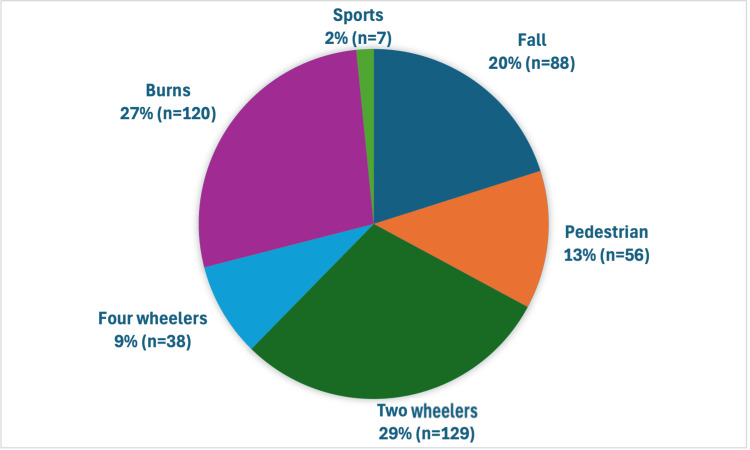
Mode of injury among pediatric trauma cases Pie chart illustrating the distribution of trauma mechanisms in the study cohort (n=438). Two-wheeler accidents accounted for the highest proportion (29%), followed by burns (27%), falls from height (20%), pedestrian injuries (13%), four-wheeler accidents (9%), and sports-related trauma (2%). Together, two-wheeler and burn injuries comprised over half of all cases, highlighting key targets for preventive intervention.

We found that 62.7% of patients presented with simple injuries. Roads emerged as the most common site of trauma (63%), followed by homes and public spaces. We recorded two-wheeler accidents (29.5%), burns (27.4%), and falls from height (20.1%) as the leading mechanisms of injury. Figure 3 visualizes the distribution of trauma mechanisms, and Table 1 further stratifies these by age group.

Patients most frequently reported pain (50.37%), followed by tenderness (13.97%), vomiting (7.73%), and loss of consciousness (5.2%). Table 2 summarizes the symptomatology profile across injury presentations.

**Table 2:** Symptomatology profile of patients included in the study (n=673) Although 438 children were enrolled, the total number of injuries recorded was 673. This discrepancy reflects the fact that individual patients often sustained multiple injuries affecting different anatomical regions. Each injury was accounted for separately to better understand trauma distribution and referral needs.

Symptomatology	Total (%)
Pain	339 (50.37%)
Bleeding	46 (6.84%)
Decreased range of motion	6 (0.89%)
Loss of consciousness	35 (5.2%)
Tenderness	94 (13.97%)
Swelling	19 (2.82%)
Vomiting	52 (7.73%)
Giddiness	17 (2.53%)
Injury site	24 (3.57%)
Burning sensation	17 (2.53%)
Irritability	2 (0.3%)
Deformity	9 (1.34%)
Seizures	13 (1.93%)
n-value	673

We observed that abrasions (41.55%) and lacerations (30.14%) were the most common wound types. The upper limb (22.81%), head (21.86%), and lower limb (20.77%) were the most frequently affected anatomical regions. Table 1 details these distributions. We referred patients most often to orthopedics (72.88%), followed by pediatrics (9.48%) and ENT/ophthalmology (5.88% each), as shown in Table 3.

**Table 3:** Departments consulted for patient referrals in the study population (n=306) Only patients who received specialist consultations beyond emergency care were included. Those managed solely by the emergency team or discharged without referral were excluded from this count.

Consultant department	Total (%)
Orthopedics	223 (72.88%)
Otolaryngology	18 (5.88%)
Ophthalmology	18 (5.88%)
Pediatrics	29 (9.48%)
Dermatology	1 (0.33%)
Dentistry	9 (2.94%)
Plastic surgery	6 (1.96%)
Gynecology	1 (0.33%)
Neurology	0 (0%)
Cardiology	1 (0.33%)
Thoracic surgery	0 (0%)
n-value	306

We performed radiological investigations in 335 cases. X-ray was the most commonly used modality (64.48%), followed by computed tomography at 34.33% and ultrasonography at 0.9%. Table 4 outlines the distribution of imaging modalities.

**Table 4:** Distribution of radiological examinations among the study population (n=335) Radiological data reflect cases where imaging was performed, excluding minor injuries or clinically evident diagnoses that did not require imaging.

Radiological examination	Total (%)
X-ray	216 (64.48%)
Computed tomography	115 (34.33%)
Ultrasonography	3 (0.9%)
Electrocardiogram	1 (0.3%)
n-value	335

We managed most cases conservatively (94.75%), with only 5.25% requiring surgical intervention. Table 5 summarizes the therapeutic approaches administered across the cohort.

**Table 5:** Distribution of therapeutic interventions among the study population (n=438) Therapeutic interventions were categorized as either conservative or surgical. Conservative management included observation, medication, wound care, and non-invasive procedures. Surgical interventions were recorded only when operative treatment was performed during the hospital stay.

Intervention	Total (%)
Conservative	415 (94.75%)
Surgical	23 (5.25%)
n-value	438

## Discussion

Our study shows that road traffic accidents, burns, and falls remain the most significant contributors to pediatric trauma in South India. Thakur et al. [4] similarly reported road traffic accidents as the dominant mechanism among children in a level I trauma center, with two-wheelers particularly implicated. Kundal et al. [5] reinforced this pattern in urban Indian settings, highlighting the disproportionate burden of vehicular trauma among adolescents. Peden et al. [6] emphasized transport-related injuries as a leading cause of morbidity and mortality in children, particularly in low- and middle-income countries. The World Health Organization's global report further underscores the urgency of road safety enforcement [2]. Newberry et al. [7] demonstrated that pediatric use of emergency medical services in India remains under-optimized, with delayed transport and triage contributing to poor outcomes.

Our data show that 63% of injuries occurred on roads, with two-wheeler accidents accounting for 29.5% of cases, underscoring the need for targeted interventions. Unlike prior studies focused on urban trauma centers, our cohort from Villupuram captures a semi-urban population, revealing that road traffic accidents dominate pediatric trauma even outside metropolitan zones.

This pattern may reflect local contextual factors such as limited traffic enforcement, poor road safety infrastructure, and delayed emergency transport, which are common challenges in semi-urban Tamil Nadu. Inadequate pedestrian pathways, lack of child-specific road safety education, and high two-wheeler density without proportional helmet use may further contribute to the observed injury burden. These systemic gaps highlight the need for targeted, region-specific interventions.

Consistent with prior findings [4], our cohort showed a strong male predominance (71%), with statistically significant variation across age groups (χ²=32.86; p<0.001). This likely reflects increased outdoor exposure and risk-taking behavior among boys. Studies from Pakistan and Nepal have similarly shown that boys are more likely to be injured as road users or during unsupervised mobility [8-10]. Our data reinforce this pattern, with adolescent males (10-18 years) accounting for over 70% of male trauma presentations.

Burn injuries emerged as the second most common mechanism (27.4%), with clear age-related variation. Scalds predominated in younger children, while flame burns were more frequent in older pediatric age groups. Bakhiet et al. [11] and Asefa et al. [12] reported similar patterns in Sudan and Ethiopia. Toma et al. [13] extended this trend across Romania, highlighting the universal vulnerability of children to domestic burn hazards. Ebrahem et al. [14] and Sharma et al. [15] emphasized the role of unsafe cooking environments and caregiver inattention in burn-related morbidity, particularly in low-income households. Our data show burns were most frequent among adolescents (n=90), but also notable in the 0-3 age group (n=12), suggesting dual peaks in domestic and activity-related exposures.

Falls were the third most frequent mechanism (20.1%). Patel et al. [16] demonstrated that falls significantly contributed to pediatric trauma mortality in urban India, particularly among children with isolated head trauma. Manoj et al. [3] added environmental and socioeconomic context, identifying unprotected rooftops, unsafe staircases, and limited caregiver supervision as major risk factors. Ma et al. [17] applied Bronfenbrenner's ecological theory to show how household-level factors, such as cluttered spaces and lack of safety gates, contribute to fall injuries. Selvakumar et al. [18] and Inbaraj et al. [19] emphasized the need for caregiver education and community-based injury awareness programs, aligning with our findings from home-based injuries (26.9%).

Regarding long-term outcomes, Subba Rao et al. [20] conducted a five-year follow-up study and demonstrated persistent neurological and psychosocial sequelae in children with head injuries despite initial survival. Anderson et al. [21] reinforced this concern, showing that pediatric traumatic brain injury often leads to chronic cognitive and emotional deficits. Jagnoor et al. [22] emphasized the importance of neurorehabilitation and quality-of-life monitoring, while Kassam-Adams and Butler [23] advocated for trauma-informed care models. Desai et al. [24] and Satapathy and Walia [25] highlighted the psychosocial toll of trauma on children and the need for integrated mental health support.

When compared internationally, Amato et al. [26] showed that pediatric trauma mortality in India remained significantly higher than in the United States, even after adjusting for injury severity. This disparity underscores systemic challenges in prehospital care, timely diagnosis, and access to advanced trauma interventions. Shetty et al. [27] called for pediatric emergency medicine training programs in India, while Ezhumalai et al. [28] demonstrated that referral education modules can improve triage efficiency. Bhalla et al. [29] revealed that layperson transport systems and delayed emergency medical services response times remain major barriers to survival. Rickenbach et al. [30] confirmed that prolonged prehospital time correlates with increased mortality in pediatric trauma, reinforcing the urgency of system-level reforms.

Limitations

This study has several limitations. We conducted it at a single tertiary care center using purposive sampling, which may restrict generalizability to other regions or community-level settings. The six-month study period precluded analysis of seasonal variation, which could influence injury patterns. We excluded cases with incomplete records, potentially introducing selection bias.

Importantly, our study design likely underrepresents minor pediatric injuries that are either managed at home or treated in primary care settings. As a result, the sample is skewed toward moderate and severe trauma cases that warrant tertiary-level intervention. This selection bias limits our ability to capture the full spectrum of pediatric injuries in the community, particularly those with low-acuity or self-limiting presentations. Additionally, we assessed only short-term clinical outcomes and did not evaluate long-term functional, neurological, or psychosocial sequelae. Despite these constraints, our findings offer valuable insights into pediatric trauma in a semi-urban Indian context, where epidemiological data remain scarce.

## Conclusions

Our study demonstrates that pediatric trauma in Villupuram is dominated by road traffic accidents, burns, and falls, with adolescent males most frequently affected. Abrasions and lacerations were the most common injuries, while extremities and head regions were most involved. These findings align with broader trends in South Asia and highlight systemic challenges in trauma care, particularly when compared internationally.

Based on these findings, we recommend regionally relevant preventive strategies such as enforcing mandatory child helmet laws and two-wheeler safety regulations, implementing caregiver-focused burn safety programs to promote safe cooking practices, and integrating school-based injury-prevention curricula to build early safety awareness. Fall-prevention efforts should include safer housing design, railing installation, and community education. Strengthening prehospital care systems, standardizing multidisciplinary trauma protocols, and establishing long-term rehabilitation services are essential to address both physical and psychosocial sequelae. Finally, we advocate for the creation of regional pediatric trauma registries to inform national policy and reduce the overall burden of childhood trauma. Future studies should focus on long-term functional and neurocognitive outcomes and explore scalable, community-based prevention strategies.
